# Associations between microRNA (miR-25, miR-32, miR-125, and miR-222) polymorphisms and recurrent implantation failure in Korean women

**DOI:** 10.1186/s40246-019-0246-y

**Published:** 2019-12-16

**Authors:** Jeong Yong Lee, Eun Hee Ahn, Jung Oh Kim, Han Sung Park, Chang Soo Ryu, Ji Hyang Kim, Young Ran Kim, Woo Sik Lee, Nam Keun Kim

**Affiliations:** 1 Department of Biomedical Science, College of Life Science, CHA University, Seongnam, 13488 South Korea; 2 Department of Obstetrics and Gynecology, CHA Bundang Medical Center, School of Medicine, CHA University, Seongnam, 13496 South Korea; 3 Department of Obstetrics and Gynecology, CHA Gangnam Medical Center, School of Medicine, CHA University, Seoul, 06135 South Korea

**Keywords:** MicroRNA, Polymorphism, Recurrent implantation failure, Single nucleotide variation

## Abstract

**Background:**

Recurrent implantation failure (RIF) is the failure of embryos to implant more than two times in a given individual. There is debate about a precise definition for RIF, but we consider more than two implantation failures for individuals who undergo in vitro fertilization-embryo transfer (IVF-ET) to constitute RIF. There are many potential reasons for RIF, including embryonic factors, immunological factors, uterine factors, coagulate factors, and genetic factors. Genetic variation has been suggested as one of the contributing factors leading to RIF, and a number of single-nucleotide polymorphisms (SNPs) have been reported to be associated with RIF. The recent elucidation of miRNA functions has provided new insight into the regulation of gene expression.

**Methods:**

We investigated associations between polymorphisms in four miRNAs and RIF in 346 Korean women: 118 patients with RIF and 228 controls. We determined the genotypes of the miRNAs in the study participants by polymerase chain reaction-restriction fragment-length polymorphism (PCR-RFLP) analysis. We analyzed the effects of genotypes, allele combinations, and environmental and clinical factors on the risk of RIF.

**Results:**

The *miR*-25 T/*miR*-125aT/*miR*-222G (odds ratio (OR), 0.528; 95% confidence interval (CI), 0.282–0.990; *P* = 0.044) and *miR*-25 T/*miR*-125aT allele combinations were associated with a reduced risk of RIF. The *miR*-25 T/*miR*-32C/*miR*-125aC/*miR*-222 T allele combination was associated with an increased risk of RIF. The *miR*-222GT+TT genotypes interacted with high prothrombin time (≥ 12 s) to increase the risk of RIF.

**Conclusions:**

MicroRNA polymorphisms are significantly different between patients that experience RIF and healthy controls. Combinations of microRNA polymorphisms were associated with the risk of RIF. Interactions between environmental factors and genotypes increased the risk of RIF in Korean women.

## Introduction

Recurrent implantation failure (RIF) is when implanted embryos repeatedly fail. There is debate about a precise definition for RIF [1–3], but we consider more than two implantation failures for individuals who undergo in vitro fertilization-embryo transfer (IVF-ET) to constitute RIF. Many potential reasons for RIF have been reported, including embryonic factors, immunological factors, uterine factors, coagulate factors, and genetic factors.

MicroRNAs (miRNAs) are small (approximately 18–22 nucleotides), non-protein-coding RNAs that regulate gene expression by causing transcript degradation and translational repression [4, 5]. Cellular processes, such as proliferation and apoptosis, are also regulated by miRNAs via complementary base-pair binding to 3’UTR regions of mRNAs, resulting in mRNA degradation and translational repression [4]. One study that evaluated microRNA expression in patients with RIF discovered that 3800 genes could be regulated by only 13 microRNAs [5]. Another study reported that 313 genes were upregulated or downregulated in patients with RIF [6]. A study of IVF in rodents revealed that downregulation of the microRNA 199a-5p was associated with poor blastocyst development [7].

Maternal vascular development is very important in early pregnancy [8]. Angiogenesis plays a key role in corpus luteum development, embryo implantation, and placentation [8]. Because both angiogenesis and vasculogenesis are essential during early pregnancy for the proper conditioning of the uterus and placenta, high expression levels of VEGF and KDR are required [9]. Growth factors such as VEGF and their respective receptors drive the angiogenic process [8, 9], while other proteins, such as fibrinogen, also have an effect [8].

VEGF functions to increase cell proliferation, migration, and differentiation and is highly polymorphic [10]. VEGF has been associated with RIF after IVF-ET with intracytoplasmic sperm injection [11]. Abnormal maternal expression of VEGF may be associated with abnormal angiogenesis during implantation [12]. A previous study suggested that miRNAs regulate VEGF expression [13], implicating miRNAs as a possible cause of implantation failure [14]. KDR is a VEGF receptor that plays an essential role in angiogenesis in the placenta and fetus [15]. Additionally, in vivo and in vitro studies showed that KDR disruption can cause defects in fetal development and angiogenesis [16]. During pregnancy, following fibrinogen-fibrin conversion, the levels of thrombin and markers of coagulation and fibrinolytic activation are increased [16, 17].

Research shows that miRNAs affecting the expression of certain genes are associated with various diseases [18, 19] as well as with ovarian function [20, 21]. A recent study reported that miRNAs are involved in the uterine condition and affect other stages of pregnancy, such as implantation [22]. These findings suggest other possibly important roles of miRNAs in reproduction. We selected four miRNA polymorphisms (miR-25 rs1527423 T>C, miR-32 rs7041716 C>A, miR-125a rs12976445 C>T, and miR-222 rs34678647 G>T) that were previously shown to impact the regulation of genes related to vascular function, thrombosis, and angiogenesis, all of which have been shown to be associated with unexplained female infertility [23, 24]. Each of these selected polymorphisms occurs in miRNAs that bind to the 3’UTRs of VEGF, KDR, and fibrinogen mRNAs. Thus, we assessed the frequencies of the four miRNA polymorphisms in Korean women and analyzed their associations with the risk of RIF.

## Results

We investigated the relationships between polymorphisms in each miRNA (*miR-25* T>C, *miR-32*C>A, *miR-125*C>T, and *miR-222*G>T) and RIF in Korean women. The demographic clinical profiles of the participants are presented in Table 1. The controls and the patients with RIF were matched by age and gender. The PT, aPTT, and total cholesterol levels were significantly higher (*P* < 0.05) in the controls than in the patients with RIF. The genotype frequencies of the patients and controls are shown in Table 2. There were no significant differences in genotype frequencies between the patients and the controls. The reference genotypes of miR-25, miR-32, miR-125a, and miR-222 were present in 78.5%, 82.0%, 74.6%, and 58.3% of the controls and 79.7%, 79.7%, 83.1%, and 52.5% of the patients with RIF, respectively. The genotypes of all four miRNAs were in HWE.

**Table 1:** Clinical profiles of patients with RIF and controls individuals

Characteristics	Controls (*n* = 228)	RIF (*n* = 118)	*P* value
Age (years)	33.8 ± 3.26	34.1 ± 3.21	0.296
BMI (kg/m^2^)	21.7 ± 3.31	21.0 ± 2.76	0.084
Previous implantation failure (n)	0	112	N/A
Live births (n)	1.77	0	N/A
Mean gestational age (weeks)	39.2 ± 1.69	0	N/A
PT (s)	11.3 ± 3.12	11.1 ± 1.49	**0.0003**^**†**^
aPTT (s)	30.8 ± 4.62	29.2 ± 3.42	**0.024**^**†**^
PLT (10^3^/μl)	243.3 ± 59.99	233.9 ± 58.49	0.186
Homocysteine (μmol/L)	N/A	6.6 ± 1.34	N/A
Folate (mg/ml)	15.0 ± 8.97	15.6 ± 11.29	0.865
Total cholesterol (mg/dl)	229.4 ± 90.57	189.1 ± 44.68	0.062^†^
Uric acid (mg/dl)	N/A	3.9 ± 0.96	N/A
BUN (mg/dl)	8.1 ± 1.83	10.3 ± 2.82	**< 0.0001**^**†**^
Creatinine (mg/dl)	0.7 ± 0.08	0.8 ± 0.10	**< 0.0001**
E2 (Basal)	26.3 ± 14.72	2465.2 ± 2457.73	**< 0.0001**^**†**^
TSH (mU/L)	N/A	2.3 ± 1.43	N/A
FSH (mU/L)	8.2 ± 2.85	8.7 ± 4.52	0.784†
LH (mU/L)	3.3 ± 1.76	4.8 ± 2.29	**< 0.0001**^**†**^
Prolactin (ng/mL)	N/A	12.6 ± 6.21	N/A
WBC (10^3^/μl)	N/A	7.2 ± 2.86	N/A
Hgb (g/dl)	N/A	12.6 ± 1.41	N/A
CD3 (pan T)	N/A	66.2 ± 11.28	N/A
CD4 (helper T)	N/A	33.9 ± 8.68	N/A
CD8 (suppressor)	N/A	29.0 ± 7.85	N/A
CD19 (B-cell)	N/A	11.0 ± 4.45	N/A
CD56 (NK cell)	N/A	20.0 ± 9.56	N/A

Italicized text indicates significant *P* values. Data are presented as the mean ± standard deviation

*RIF*, recurrent implantation failure; *BMI*, body mass index; *PT*, prothrombin time; *aPTT*, activated partial thromboplastin time; *PLT*, platelet count; *BUN*, blood urea nitrogen; *E2*, estradiol; *TSH*, thyroid stimulating hormone; *FSH*, follicle-stimulating hormone; *LH*, luteinizing hormone; *WBC*, white blood cell; *Hgb*, hemoglobin

^†^Mann-Whitney test

**Table 2:** Comparison of genotype frequencies of miRNA polymorphisms between patients and controls

Genotypes	Controls (*n* = 228)	IF ≥ 2 (*n* = 118)	AOR (95% CI)^a^	*P*^a^	IF ≥ 3 (*n* = 106)	AOR (95% CI)^a^	*P*^a^	IF ≥ 4 (*n* = 75)	AOR (95% CI)^a^	*P*^a^
miR-25 rs1527423 T>C										
TT	179 (78.5)	94 (79.7)	1.000 (reference)		84 (79.25)	1.000 (reference)		57 (76.00)	1.000 (reference)	
TC	46 (20.2)	23 (19.5)	0.947 (0.541–1.658)	0.849	21 (19.81)	0.968 (0.542–1.728)	0.913	17 (22.67)	1.146 (0.608–2.159)	0.673
CC	3 (1.3)	1 (0.8)	0.632 (0.065–6.169)	0.693	1 (0.94)	0.709 (0.073–6.931)	0.767	1 (1.33)	1.044 (0.106–10.249)	0.971
Dominant (TT vs TC+CC)			0.927 (0.535–1.606)	0.788		0.952 (0.540–1.679)	0.865		1.139 (0.613–2.116)	0.680
Recessive (TT+TC vs CC)			0.639 (0.066–6.218)	0.700		0.713 (0.073–6.959)	0.771		1.014 (0.104–9.927)	0.991
HWE *P*	0.981	0.753			0.804			0.832		
miR-32 rs7041716 C>A										
CC	187 (82.0)	94 (79.7)	1.000 (reference)		85 (80.19)	1.000 (reference)		58 (77.33)	1.000 (reference)	
CA	39 (17.1)	22 (18.6)	1.123 (0.629–2.004)	0.695	19 (17.92)	1.069 (0.582–1.963)	0.829	16 (21.33)	1.315 (0.683–2.530)	0.413
AA	2 (0.9)	2 (1.7)	1.893 (0.262–13.686)	0.527	2 (1.89)	2.072 (0.286–15.003)	0.471	1 (1.33)	1.446 (0.128–16.310)	0.766
Dominant (CC vs CA+AA)			1.163 (0.663–2.040)	0.600		1.121 (0.623–2.016)	0.704		1.322 (0.697–2.508)	0.392
Recessive (CC+CA vs AA)			1.876 (0.260–13.511)	0.532		2.064 (0.286–14.894)	0.472		1.390 (0.124–15.610)	0.790
HWE *P*	0.983	0.594			0.45			0.906		
miR-125a rs12976445 C>T										
CC	170 (74.6)	98 (83.1)	1.000 (reference)		87 (82.08)	1.000 (reference)		63 (84.00)	1.000 (reference)	
CT	52 (22.8)	20 (16.9)	0.680 (0.383–1.208)	0.188	19 (17.92)	0.734 (0.408–1.321)	0.303	12 (16.00)	0.633 (0.316–1.266)	0.196
TT	6 (2.6)	0 (0.0)	N/A	0.995	0 (0.00)	0.000 (0.000–0.000)	0.995	0 (0.00)	0.000 (0.000–0.000)	0.993
Dominant (CC vs CT+TT)			0.610 (0.346–1.075)	0.087		0.658 (0.368–1.177)	0.158		0.568 (0.286–1.130)	0.107
Recessive (CC+CT vs TT)			N/A	0.995		N/A	0.995		N/A	0.993
HWE *P*	0.408	0.315			0.311			0.448		
miR-222 rs34678647 G>T										
GG	133 (58.3)	62 (52.5)	1.000 (reference)		56 (52.83)	1.000 (reference)		39 (52.00)	1.000 (reference)	
GT	79 (34.6)	48 (40.7)	1.320 (0.825–2.112)	0.247	42 (39.62)	1.284 (0.787–2.096)	0.317	31 (41.33)	1.343 (0.775–2.327)	0.294
TT	16 (7.0)	8 (6.8)	1.030 (0.415–2.560)	0.949	8 (7.55)	1.144 (0.457–2.859)	0.774	5 (6.67)	0.999 (0.340–2.938)	0.999
Dominant (GG vs GT+TT)			1.273 (0.813–1.993)	0.291		1.262 (0.792–2.009)	0.327		1.288 (0.761–2.179)	0.346
Recessive (GG+GT vs TT)			0.944 (0.391–2.279)	0.898		1.054 (0.435–2.553)	0.908		0.911 (0.321–2.589)	0.861
HWE *P*	0.371	0.752			0.974			0.726		

*IF*, implantation failure; *AOR*, adjusted odds ratio; *for AOR*, OR adjusted by age of participants; *95% CI*, 95% confidence interval; *N/A*, not applicable

^a^Adjusted by age

To identify associations between allele combinations and RIF risk, we analyzed combinations of four polymorphisms of the miRNA genes (Table 3). The combinations *miR-25* T/*miR-125a*T/*miR-222*G (AOR, 0.528; 95% CI, 0.282–0.990; *P* = 0.044) and *miR-25* T/*miR-125a*T (AOR, 0.510; 95% CI, 0.285–0.913; *P* = 0.022) were associated with a lower risk of RIF. By contrast, the combinations *miR*-*25* T/*miR*-*32*C/*miR-125a*C/*miR-222* T (AOR, 1.496; 95% CI, 1.000–2.237; *P* = 0.049) and *miR-25* T/*miR-32*C/*miR-222* T (AOR, 1.585; 95% CI, 1.071–2.345; *P* = 0.021) were associated with a greater risk of RIF.

**Table 3:** Allele combination analysis for the miRNA polymorphisms in patients and controls using MDR

Allele combinations	Overall (2n = 692)	Controls (2n = 456)	RIF (2n = 236)	OR (95% CI)	*P*^a^
miR-25 T>C/ miR-32 C>A/ miR-125aC>T/ miR-222G>T					
T-C-C-G	0.5035	0.5203	0.4841	1.000 (reference)	
T-C-C-T	0.2081	0.1798	0.2482	1.496 (1.000–2.237)	**0.049**
T-C-T-G	0.0884	0.1026	0.0566	0.575 (0.299–1.106)	0.094
T-C-T-T	0.0047	0.0059	0.0065	1.386 (0.228–8.414)	0.662
T-A-C-G	0.0563	0.0436	0.0797	1.975 (1.014–3.847)	**0.042**
C-C-C-G	0.0653	0.0527	0.0753	1.559 (0.813–2.989)	0.178
C-C-C-T	0.0124	0.0260	0.0000	0.083 (0.005–1.415)	**0.022**
C-C-T-G	0.0136	0.0140	0.0191	1.732 (0.518–5.798)	0.352
miR-25 T>C/ miR-125aC>T/ miR-222G>T					
T-C-G	0.5598	0.5617	0.5635	1.000 (reference)	
T-C-T	0.2253	0.2013	0.2650	1.318 (0.899–1.933)	0.157
T-T-G	0.0949	0.1120	0.0594	0.528 (0.282–0.990)	**0.044**
T-T-T	0.0087	0.0110	0.0062	0.385 (0.045–3.331)	0.668
C-C-G	0.0783	0.0691	0.0868	1.203 (0.662–2.185)	0.543
C-C-T	0.0152	0.0276	0.0000	0.071 (0.004–1.207)	**0.006**
C-T-G	0.0141	0.0138	0.0191	1.604 (0.481–5.355)	0.523
C-T-T	0.0037	0.0036	0.0000	0.384 (0.018–8.068)	0.550
miR-25 T>C/ miR-32 C>A/ miR-222 G>T					
T-C-G	0.5909	0.6224	0.5405	1.000 (reference)	
T-C-T	0.2128	0.1841	0.2554	1.585 (1.071–2.345)	**0.021**
T-A-G	0.0643	0.0538	0.0824	1.686 (0.896–3.173)	0.102
T-A-T	0.0207	0.0256	0.0158	0.740 (0.234–2.338)	0.785
C-C-G	0.0788	0.0665	0.0940	1.627 (0.903–2.931)	0.103
C-C-T	0.0178	0.0327	0.0000	0.071 (0.004–1.204)	**0.007**
C-A-G	0.0131	0.0139	0.0119	1.109 (0.273–4.507)	1.000
C-A-T	0.0016	0.0010	0.0000	N/A	N/A
miR-25 T>C/ miR-222G>T					
T-G	0.6545	0.6739	0.6229	1.000 (reference)	
T-T	0.2342	0.2121	0.2712	1.378 (0.950–1.999)	0.090
C-G	0.0926	0.0827	0.1059	1.374 (0.799–2.362)	0.249
C-T	0.0187	0.0313	0.0000	0.072 (0.004–1.214)	**0.007**
miR-25 T>C/ miR-125aC>T					
T-C	0.7844	0.7625	0.8277	1.000 (reference)	
T-T	0.1043	0.1235	0.0663	0.510 (0.285–0.913)	**0.022**
C-C	0.0942	0.0972	0.0875	0.852 (0.492–1.474)	0.566
C-T	0.0170	0.0169	0.0184	0.892 (0.265–3.002)	1.000

Italicized text indicates significant *P* values

*RIF*, recurrent implantation failure; *OR*, odds ratio; *95% CI*, 95% confidence interval; *N/A*, not applicable; *MDR*, multi-dimensional reduction

^a^Fisher’s exact test

We analyzed the interactions between coagulation factors and the miRNAs (Table 4). There were significant differences in PT, aPTT, uric acid, BUN, creatinine, and LH levels between RIF and control patients (*P* < 0.05 for each comparison). We looked for synergistic interactions between miRNA polymorphisms and environmental factors. The *miR-222*GT+TT genotypes interacted significantly with blood coagulation factors to increase the risk of RIF (*P* < 0.05); individuals with PT in the upper quartile (PT ≥ 12 s) and those with the *miR-222*GT+TT genotypes had a dramatically increased risk of RIF.

**Table 4:** Interaction analysis between recurrent implantation failure and coagulation factors in participants

	miR-25 rs1527423 T > C		miR-32 rs7041716 C > A		miR-125a rs12976445 C > T		miR-222 rs34678647 G > T	
PT (s)	TT	TC + TT	CC	CA + CC	TT	CT + TT	TT	GT + TT
<12	1.000 (reference)	0.935 (0.526–1.665)	1.000 (reference)	1.433 (0.808–2.540)	1.000 (reference)	0.544 (0.295–1.005)	1.000 (reference)	1.135 (0.706–1.824)
≥12	**7.560 (2.409–23.725)**	N/A	**13.175 (3.745–46.350)**	N/A	**5.946 (1.864–18.971)**	N/A	**5.515 (1.056–28.792)**	**13.269 (2.823–62.379)**
aPTT (s)								
<33.8	1.000 (reference)	1.022 (0.578–1.808)	1.000 (reference)	1.211 (0.678–2.161)	1.000 (reference)	0.547 (0.295–1.016)	1.000 (reference)	1.108 (0.693–1.771)
≥33.8	1.574 (0.659–3.760)	0.440 (0.050–3.865)	1.393 (0.598–3.245)	0.957 (0.085–10.779)	1.103 (0.415–2.931)	1.242 (0.339–4.558)	0.621 (0.193–1.998)	**5.123 (1.379–19.030)**
PLT (×10^3^/μl)								
<294	1.000 (reference)	0.741 (0.391–1.403)	1.000 (reference)	1.081 (0.573–2.037)	1.000 (reference)	0.629 (0.340–1.163)	1.000 (reference)	**1.386 (0.853–2.254)**
≥294	0.832 (0.375–1.845)	1.641 (0.610–4.420)	0.999 (0.471–2.121)	1.512 (0.506–4.521)	1.167 (0.571–2.384)	0.616 (0.161–2.360)	1.397 (0.578–3.380)	1.114 (0.445–2.791)

Italicized text indicates significant *P* values. The upper quartile for PT, aPTT, and PLT was 12 s, 33.8 s, and 294 × 10^3^ platelets/μl respectively

*AOR*, adjusted odds ratio; *aPTT*, activated partial thromboplastin time; *PLT*, platelet count; *PT*, prothrombin time

Data are expressed as AOR (95% CI)

We performed ANOVA for each group between miRNA genotypes and the clinical parameters. As shown in Table 5, creatinine levels decreased when *miR-222* polymorphisms changed in control women (*P* < 0.05). Furthermore, FSH levels were shown to increase with specific *miR-222* polymorphisms in RIF patients (Table 6). Therefore, creatinine and FSH levels may be dependent on *miRNA-222* polymorphisms.

**Table 5:** Differences in various clinical parameters according to micro RNA polymorphisms in control women

Control														
Genotypes	Age (years)	BMI (kg/m2)	Live births (*n*)	Mean gestational age (weeks)	PT (s)	aPTT (s)	PLT (10^3^/μl)	Folate (mg/ml)	Total cholesterol (mg/dl)	BUN (mg/dl)	Creatinine (mg/dl)	E2 (Basal)	FSH (mU/L)	LH (mU/L)
	Mean ± SD (228)	Mean ± SD (104)	Mean ± SD (74)	Mean ± SD (96)	Mean ± SD (47)	Mean ± SD (77)	Mean ± SD (187)	Mean ± SD (14)	Mean ± SD (9)	Mean ± SD (30)	Mean ± SD (30)	Mean ± SD (109)	Mean ± SD (109)	Mean ± SD (106)
miR-25 T>C														
TT	33.8 ± 3.30	21.7 ± 3.41	1.7 ± 0.62	39.1 ± 1.75	11.7 ± 3.62	30.9 ± 4.76	243.8 ± 59.58	16.1 ± 10.79	188.4 ± 40.62	8.0 ± 1.74	0.7 ± 0.09	27.0 ± 15.02	8.1 ± 2.89	3.1 ± 1.55
TC	33.8 ± 3.20	22.0 ± 2.91	2.0 ± 0.37	39.4 ± 1.47	10.4 ± 0.98	30.5 ± 4.31	238.0 ± 60.87	13.1 ± 4.61	373.0 ± 52.33	8.1 ± 2.34	0.7 ± 0.05	24.0 ± 14.38	8.5 ± 2.90	4.0 ± 2.17
CC	33.3 ± 2.52	N/A	N/A	N/A	N/A	N/A	297.7 ± 57.76	N/A	N/A	N/A	N/A	22.2 ± 7.87	7.2 ± 1.26	5.6 ± 1.17
*P* value	0.974	0.707	0.077	0.465	0.208	0.739	0.246	0.563	**0.001**	0.915	0.316	0.608	0.681	**0.006**
miR-32 C>A														
CC	33.7 ± 3.28	21.7 ± 3.33	1.8 ± 0.56	39.5 ± 1.54	11.4 ± 3.24	30.9 ± 4.60	242.0 ± 61.76	15.1 ± 9.34	235.9 ± 102.77	8.3 ± 1.79	0.7 ± 0.07	27.0 ± 14.82	8.0 ± 2.72	3.2 ± 1.67
CA	33.9 ± 3.30	22.0 ± 3.34	1.9 ± 0.70	37.8 ± 1.86	10.1 ± 1.84	29.3 ± 4.75	249.8 ± 51.16	14.9	207.0 ± 31.11	6.9 ± 1.66	0.7 ± 0.10	24.0 ± 14.35	8.9 ± 3.34	3.8 ± 2.11
AA	35.0	22.9	1.0	39.5 ± 0.71	11.4	35.7	250.5 ± 67.18	N/A	N/A	N/A	N/A	11.1	5.7	4.1
*P* value	0.809	0.882	0.310	**0.002**	0.720	0.341	0.796	0.987	0.718	0.092	0.316	0.430	0.291	0.296
miR-125a C>T														
CC	33.8 ± 3.39	21.7 ± 3.19	1.8 ± 0.61	39.2 ± 1.75	11.5 ± 3.59	30.5 ± 4.09	240.8 ± 60.91	15.1 ± 9.91	238.2 ± 86.80	7.8 ± 1.84	0.7 ± 0.09	26.5 ± 14.86	8.0 ± 2.43	3.3 ± 1.78
CT	33.6 ± 2.83	21.4 ± 3.70	1.6 ± 0.51	39.2 ± 1.52	10.9 ± 0.56	31.5 ± 6.91	247.1 ± 58.14	15.0 ± 5.62	212.0 ± 115.31	9.3 ± 1.33	0.7 ± 0.04	25.0 ± 14.57	8.5 ± 3.83	3.4 ± 1.78
TT	33.3 ± 3.61	23.7 ± 4.04	1.8 ± 0.50	38.3 ± 0.87	10.4 ± 0.76	32.2 ± 0.67	276.5 ± 45.14	0.0	N/A	N/A	N/A	37.5 ± 12.09	8.0 ± 2.97	2.0 ± 0.42
*P* value	0.863	0.460	0.573	0.525	0.791	0.673	0.330	0.988	0.711	0.098	**< 0.001**	0.506	0.755	0.554
miR-222 G>T														
GG	33.6 ± 3.17	21.5 ± 3.24	1.7 ± 0.58	39.0 ± 1.45	11.0 ± 1.90	31.2 ± 5.25	243.5 ± 58.97	17.1 ± 11.22	177.4 ± 44.07	8.4 ± 1.65	0.7 ± 0.06	25.6 ± 14.52	8.0 ± 2.38	3.4 ± 1.54
GT	33.9 ± 3.40	22.0 ± 3.47	1.8 ± 0.61	39.5 ± 1.74	10.8 ± 0.72	29.8 ± 3.38	240.9 ± 62.74	12.3 ± 4.13	294.5 ± 95.55	7.8 ± 1.80	0.6 ± 0.08	28.1 ± 15.28	8.4 ± 3.54	3.2 ± 2.08
TT	34.4 ± 3.44	22.0 ± 3.53	2.2 ± 0.45	38.9 ± 2.93	16.5 ± 11.14	31.1 ± 2.72	254.2 ± 56.51	N/A	N/A	4.2	0.6	22.6 ± 13.98	7.9 ± 2.55	3.3 ± 1.76
*P* value	0.541	0.778	0.227	0.144	0.946	0.479	0.751	0.561	**0.050**	0.059	**0.005**	0.531	0.778	0.845

Italicized text indicates significant *P* values

*BMI*, body mass index; *PT*, prothrombin time; *aPTT*, activated partial thromboplastin time; *PLT*, platelets; *BUN*, blood urea nitrogen; *E2*, estradiol; *TSH*, thyroid stimulating hormone; *FSH*, follicle stimulating hormone; *LH*, luteinizing hormone; *WBC*, white blood cell; *Hgb*, hemoglobin

**Table 6:** Differences in various clinical parameters according to micro RNA polymorphisms in women with RIF

Patients												
Genotypes	Age (years)	BMI (kg/m^2^)	Previous implantation failure (*n*)	PT (*s*)	aPTT (*s*)	PLT (10^3^/μl)	Homocysteine (μmol/L)	Folate (mg/ml)	Total cholesterol (mg/dl)	Uric acid (mg/dl)	BUN (mg/dl)	Creatinine (mg/dl)
	Mean ± SD (118)	Mean ± SD (117)	Mean ± SD (112)	Mean ± SD (110)	Mean ± SD (110)	Mean ± SD (113)	Mean ± SD (35)	Mean ± SD (46)	Mean ± SD (47)	Mean ± SD (58)	Mean ± SD (108)	Mean ± SD (108)
miR-25 T>C												
TT	34.0 ± 3.27	21.0 ± 2.96	1.1 ± 1.55	11.1 ± 1.64	29.3 ± 3.53	230.7 ± 53.46	6.6 ± 1.41	16.1 ± 12.50	192.2 ± 48.12	3.9 ± 1.02	10.1 ± 2.95	0.8 ± 0.09
TC	34.5 ± 3.04	21.3 ± 1.78	0.9 ± 1.18	11.3 ± 0.55	28.6 ± 2.93	243.3 ± 75.15	6.4 ± 0.91	13.9 ± 4.13	177.5 ± 23.97	4.0 ± 0.74	11.2 ± 2.16	0.8 ± 0.12
CC	36.0	20.5	2.0	11.6	31.4	319.0	4.9	12.8	157.0	3.3	10.3	0.8
*P* value	0.716	0.842	0.700	0.829	0.546	0.268	0.436	0.889	0.322	0.735	0.289	0.319
miR-32C>A												
CC	34.2 ± 3.14	20.9 ± 2.69	1.2 ± 1.48	11.2 ± 1.24	29.4 ± 3.58	228.5 ± 57.00	6.5 ± 1.30	16.9 ± 11.66	188.3 ± 45.71	3.9 ± 0.99	10.2 ± 2.80	0.8 ± 0.10
CA	33.8 ± 3.61	21.3 ± 3.02	0.9 ± 1.49	10.6 ± 2.32	28.5 ± 2.50	251.4 ± 58.53	6.8 ± 1.65	9.3 ± 6.84	192.7 ± 43.39	3.8 ± 0.83	11.4 ± 2.81	0.8 ± 0.09
AA	35.0 ± 2.83	22.8 ± 4.45	0.000	10.5 ± 0.50	26.9 ± 2.97	317.0 ± 59.40	N/A	N/A	186.5 ± 4.95	0.000	8.0 ± 1.41	0.8 ± 0.07
*P* value	0.789	0.571	0.412	0.167	0.364	0.037	0.600	0.133	0.927	0.562	0.111	0.600
miR-125aC>T												
CC	34.4 ± 3.00	21.1 ± 2.69	1.0 ± 1.42	11.0 ± 1.60	28.9 ± 3.24	235.8 ± 55.54	6.5 ± 1.08	17.2 ± 12.31	188.7 ± 42.73	3.9 ± 0.94	10.2 ± 2.61	0.8 ± 0.09
CT	33.0 ± 3.94	20.7 ± 3.12	1.7 ± 1.69	11.4 ± 0.53	30.7 ± 3.97	223.8 ± 73.14	6.6 ± 2.10	10.3 ± 3.87	191.5 ± 56.72	4.2 ± 1.04	10.9 ± 3.81	0.8 ± 0.12
TT	N/A	N/A	N/A	N/A	N/A	N/A	N/A	N/A	N/A	N/A	N/A	N/A
*P* value	0.068	0.549	0.058	0.308	**0.049**	0.427	0.829	0.134	0.819	0.302	0.340	0.653
miR-222G>T												
GG	34.8 ± 3.39	21.5 ± 3.09	1.3 ± 1.82	11.1 ± 1.47	29.0 ± 3.20	231.6 ± 58.96	6.5 ± 1.39	15.9 ± 13.67	184.9 ± 48.17	4.1 ± 1.08	10.5 ± 2.71	0.8 ± 0.10
GT	33.3 ± 2.98	20.6 ± 2.34	0.8 ± 0.87	11.1 ± 1.62	29.6 ± 3.68	240.5 ± 61.10	6.8 ± 1.36	12.9 ± 5.93	195.6 ± 42.44	3.8 ± 0.77	10.0 ± 2.79	0.8 ± 0.10
TT	34.4 ± 2.13	19.5 ± 1.38	1.0 ± 1.15	11.3 ± 0.63	28.3 ± 3.43	213.6 ± 33.62	5.9 ± 0.88	26.8 ± 10.57	182.5 ± 28.95	3.0 ± 0.30	11.3 ± 3.72	0.8 ± 0.09
*P* value	0.062	0.066	0.616	0.916	0.518	0.444	0.527	0.155	0.461	0.152	0.411	0.113
E2 (Basal)	TSH (mU/L)	FSH (mU/L)	LH (mU/L)	Prolactin (ng/mL)	WBC (10^3^/μl)	Hgb (g/dl)	CD3 (panT)	CD4 (helper T)	CD8 (suppressor)	CD19 (B-cell)	CD56 (NK cell)	
Mean ± SD (51)	Mean ± SD (93)	Mean ± SD (69)	Mean ± SD (63)	Mean ± SD (94)	Mean ± SD (113)	Mean ± SD (113)	Mean ± SD (92)	Mean ± SD (92)	Mean ± SD (92)	Mean ± SD (92)	Mean ± SD (108)	
2612.3 ± 2633.01	2.3 ± 1.51	8.9 ± 4.72	4.9 ± 2.40	12.6 ± 5.67	7.2 ± 3.04	12.6 ± 1.39	66.0 ± 11.78	34.0 ± 8.72	29.0 ± 8.22	11.2 ± 4.61	19.7 ± 9.02	
1907.2 ± 1539.73	2.2 ± 1.19	8.2 ± 3.87	4.2 ± 1.75	12.9 ± 8.29	7.2 ± 2.12	12.3 ± 1.51	66.9 ± 9.31	33.3 ± 8.71	28.9 ± 6.43	10.2 ± 3.79	21.1 ± 11.58	
1500.4	1.5	6.1	5.9	11.7	9.0	12.8	N/A	N/A	N/A	N/A	N/A	
	0.838	0.703	0.406	0.962	0.833	0.530	0.765	0.745	0.950	0.373	0.558	
2472.7 ± 2463.80	2.3 ± 1.56	8.7 ± 4.59	4.8 ± 2.13	12.4 ± 6.08	7.2 ± 2.75	12.6 ± 1.40	65.9 ± 11.82	33.4 ± 8.99	28.9 ± 8.14	11.3 ± 4.89	20.3 ± 9.92	
2675.4 ± 2485.86	2.3 ± 0.80	9.1 ± 4.44	5.2 ± 2.78	14.0 ± 6.69	7.4 ± 3.50	12.5 ± 1.54	66.8 ± 9.58	35.8 ± 7.04	29.1 ± 6.70	10.1 ± 2.21	18.8 ± 8.08	
25.4 ± 28.43	1.400	5.9 ± 3.49	2.3 ± 1.77	6.100	7.1 ± 2.73	12.7 ± 0.14	70.0 ± 9.90	34.5 ± 14.85	32.5 ± 12.02	8.0 ± 1.41	22.5 ± 12.02	
0.350	0.828	0.632	0.205	0.355	0.974	0.985	0.853	0.565	0.818	0.425	0.762	
2,395.0 ± 2,202.59	2.4 ± 1.50	8.7 ± 4.65	4.8 ± 2.43	12.6 ± 6.57	7.2 ± 2.92	12.7 ± 1.38	65.3 ± 11.75	33.7 ± 8.33	28.8 ± 8.05	11.3 ± 4.54	20.4 ± 9.31	
2812.7 ± 3516.58	1.5 ± 0.68	8.7 ± 3.90	4.8 ± 1.43	12.7 ± 4.19	7.5 ± 2.63	11.9 ± 1.40	70.2 ± 7.99	34.5 ± 10.33	30.2 ± 7.02	9.9 ± 3.96	18.0 ± 10.85	
N/A	N/A	N/A	N/A	N/A	N/A	N/A	N/A	N/A	N/A	N/A	N/A	
0.730	**0.019**	0.985	0.920	0.968	0.668	**0.031**	0.106	0.732	0.501	0.260	0.342	
2475.7 ± 2224.15	2.3 ± 1.21	7.5 ± 2.58	4.5 ± 1.87	12.7 ± 6.35	7.2 ± 2.83	12.4 ± 1.43	67.2 ± 8.90	32.6 ± 8.90	29.9 ± 7.51	10.3 ± 3.74	20.6 ± 9.91	
2553.5 ± 2804.33	2.1 ± 1.22	10.1 ± 5.93	5.3 ± 2.81	12.5 ± 6.28	7.5 ± 3.06	12.7 ± 1.45	64.0 ± 15.08	35.6 ± 8.12	27.2 ± 7.75	12.7 ± 5.12	19.5 ± 9.79	
1784.6 ± 2010.09	4.0 ± 4.01	10.5 ± 5.15	4.8 ± 1.70	13.0 ± 4.88	5.8 ± 1.33	13.0 ± 0.68	68.6 ± 5.06	35.6 ± 9.09	30.3 ± 10.50	9.3 ± 4.54	18.5 ± 5.21	
0.745	0.535	**0.023**	0.540	0.984	0.329	0.255	0.762	0.273	0.283	**0.030**	0.756	

Italicized text indicates significant *P* values

*BMI*, body mass index; *PT*, prothrombin time; *aPTT*, activated partial thromboplastin time; *PLT*, platelets; *BUN*, blood urea nitrogen; *E2*, estradiol; *TSH*, thyroid stimulating hormone; *FSH*, follicle stimulating hormone; *LH*, luteinizing hormone; *WBC*, white blood cell; *Hgb*, hemoglobin

## Discussion

We examined the interplay between miRNA polymorphisms (*miR*-25 rs1527423 T>C/*miR*-32 rs7041716 C>A/*miR*-125a rs12976445 C>T/*miR*-222 rs34678647 G>T) and RIF risk. First, we compared clinical profiles between 228 healthy control individuals and 118 patients with RIF. There were significant differences in PT, aPTT, uric acid, BUN, creatinine, and LH between the patients with RIF and the controls (*P* < 0.05 for each comparison). There were no significant differences in the genotypic frequencies of any of the miRNA polymorphisms, even among subgroups, between the patients and the controls. Analysis of allele combinations among the miRNAs suggested that the combinations *miR-25* T/*miR*-*125a*T/*miR*-222G and *miR-25* T/*miR-125a*T had protective effects against RIF (*P* < 0.044 and *P* < 0.022, respectively). Also, miR-222 rs34678647 interacted significantly with blood coagulation factor (PT and aPTT) values in the upper quartile. Alteration of the functional activity of blood coagulation factors might influence implantation [25]. Several studies have suggested that thrombophilia might increase the risk of implantation failure [16].

In this study, we looked for differences in clinical parameters between RIF and control patients and also between different miRNA genotypes. We found no differences in PT, aPTT, or PLT between RIF and control patients or among the genotypes.

Many miRNAs have important roles in diverse cellular processes [23], including the regulation of molecular pathways, adhesive junctions, and cell adhesion in patients with RIF [5]. MicroRNA-181 inhibits embryo implantation in vivo by inhibiting the expression of leukemia inhibitory factor [24]. MicroRNAs play important roles in the regulation of genes that influence endometrial receptivity [26]. Several studies reported evidence that microRNAs are relevant to RIF in humans. MicroRNA-30b/d and microRNA-494 were shown to be differentially regulated in receptive endometrium [27]. Moreover, microRNA-30b was upregulated in patients with RIF [26]. Furthermore, microRNA-374 was reported to activate the Wnt/β-catenin pathway, which is associated with implantation [28]. The four miRNAs that we investigated are known to bind the 3’UTRs of fibrinogen, VEGF, and KDR [13], all of which affect implantation [11].

We predicted miRNA targets using miRBase (http://www.mirbase.org/) and TargetScan (http://www.targetscan.org/vert_72). We found that *miR*-*25* and *miR*-*32* bind fibrinogen, which plays an important role in the coagulation process [29]. The mature form of *miR-125a-3p* binds to VEGF. Altered VEGF function affects the occurrence of diseases such as breast cancer and coronary artery disease and is also associated with RPL [14, 30]. VEGF plays important roles in embryo implantation, vasculogenesis, and angiogenesis in tumor development and early gestation [8, 31]. Inactivation of a single VEGF allele in mice results in embryonic lethality and defects in several organs [15]. KDR (VEGFR2) is a VEGF receptor that may play an important role in angiogenesis and vascular development during the early stages of pregnancy [32]. The VEGF system is involved in the generation of hemangioblasts in embryos [33]. In mice, downregulation of VEGF or its two receptors results in fetal death in utero [14]. In our previous study, the VEGF and VEGFR genes were associated with an increased risk of colorectal cancer [34]. Angiogenesis may be altered via the VEGF-VEGFR pathway through decreased VEGF and VEGFR expression, resulting from an increased number of miRNA polymorphisms with high binding affinities for VEGF and VEGFR mRNAs [4, 13, 35].

In our previous study, miR-608 and miR-1302 affected the risk of RIF through their effects on coagulation factors [36]. Our present results further support the hypothesis that microRNAs associated with coagulation factors can increase the risk of RIF. In particular, prothrombin time (PT) was associated with certain microRNA polymorphisms (Table 4), suggesting that alteration of blood coagulation factors might affect implantation and also pregnancy.

A previous study reported that high FSH levels after IVF can reduce implantation rates in young women [37]. We found that the miR-222G>T mutation was associated with FSH levels (Table 6). Those results provide evidence that microRNAs can affect FSH levels, providing another potential mechanism by which microRNAs might affect implantation.

Women with RIF have significantly elevated numbers of NK cells that play a role in female reproductive performance [30, 31]. Uterine NK cells are important in pregnancy, especially in processes involving angiogenesis such as placentation [38]. In rodents, decidual natural killer (dNK) cell numbers are increased in a VEGF-abundant environment, and dNK cells express multiple angiogenic and chemokine factors in humans [39, 40]. Furthermore, dNK cells make many factors that are abundant in mammalian tissues, such as VEGF and IL-8.

Sex hormones, such as estrogen and progesterone, contribute to immune tolerance of the early pregnancy environment. Estrogen has three different forms: estrogen, estradiol (E2), and estriol. In a previous report, E2 levels were correlated with levels of IL-35, the main cytokine that maintains immune tolerance. IL-35 levels are decreased in patients with a history of recurrent spontaneous abortions [41]. E2 also stimulates CD4^+^ T cells that produce inhibitory cytokines such as IL-35, TGF-b, and IL-10 [41]. In our study, patients with RIF had higher E2 values than controls.

T regulatory (Treg) cells have potent immune-suppressive activity, and the maintenance of immune suppression is important during pregnancy [42, 43]. The numbers of CD4^+^ regulatory T cells are increased in the blood and lymph of pregnant rodents [44]. Human decidual tissue also shows increased numbers of CD4^+^ T cells [43]. Women who have experienced miscarriage have reduced numbers of Treg cells, including CD4^+^ cells, in their blood compared with women who experience healthy pregnancies.

Circulating blood VEGF levels may be linked to several NK cell variables, activated NK cells, or NK cytotoxicity [45]. The possibility of a link between NK cells and reproductive outcomes is one of the most controversial issues in reproductive medicine [46]. It is possible that elevated VEGF levels disrupt normal angiogenesis, leading to disturbed vascular architecture [39]. Women with RIF were indeed found to have significantly increased levels of VEGF in their plasma [45].

Many microRNAs are used to screen for specific diseases and also as tools for therapy selection [40]. The microRNAs used in our study might be used biomarkers to assist RIF diagnosis in the future. Pregnancy-related microRNAs might also be used in therapeutic strategies involving the addition of microRNA mimics or anti-microRNAs [47]. The development of microRNA-based therapies will require advanced drug delivery systems that can carry microparticles as a cargo [48].

There are several limitations to our study. First, it remains unclear how miRNA polymorphisms affect RIF. We only focused on SNPs in four miRNAs and tried to discover how those SNPs may interact with other factors to influence the occurrence of RIF. Many factors contribute to pregnancy failure, however, and the genotypic frequencies were not enough to explain why implantation failure occurred repeatedly. Second, our study was limited to Korean women, so results may vary in women of other nations or ethnic groups. Third, we did not examine the expression of each miRNA in tissues. It is known that miRNAs bind to the 3’UTR of their target genes to inhibit them, and each miRNA polymorphism could affect their target gene expression. Therefore, because we collected blood samples, any tissue-specific factors such as microRNA or clinical parameter that may affect RIF occurrence may not be considered. Fourth, our sample size was small, and a larger sample size is required to confirm our results. To further verify our findings, future studies should use a larger population size and include additional ethnic groups.

## Conclusion

We analyzed the association between RIF risk and miRNA (*miR-25*, *miR-32*, *miR-125*, *miR-222*) polymorphisms. The genotype frequencies of each microRNAs did not significantly differ between patients with RIF and controls, but specific allele combinations interacted with clinical parameters to increase the risk of RIF.

## Materials and Methods

### Study population

Blood samples were obtained from 118 females with RIF and 228 healthy female controls. All study samples were collected from the Department of Obstetrics and Gynecology of CHA Bundang Medical Center (Seongnam, South Korea) between March 2010 and December 2012. We defined RIF as the failure to achieve pregnancy following the completion of two fresh IVF-ET cycles with one or two good quality embryos. Each transferred embryo was cleaved into more than 10 cells. Fourteen days after, all RIF patients’ serum human chorionic gonadotrophin (hCG) concentrations were less than 5 U/ml. Individuals who were diagnosed with RIF due to anatomical, chromosomal, hormonal, infectious, autoimmune, or thrombotic causes were excluded from the study. Anatomical abnormalities were evaluated using several imaging modalities, including sonography, hysterosalpingogram, hysteroscopy, computerized tomography, and magnetic resonance imaging. Karyotyping was conducted using standard protocols to assess chromosomal abnormalities. We excluded hormonal causes of RIF, including hyperprolactinemia, luteal insufficiency, and thyroid disease, by measuring the concentrations of prolactin (PRL), thyroid-stimulating hormone (TSH), free thyroxine, follicle-stimulating hormone (FSH), luteinizing hormone (LH), estradiol (E2), and progesterone in samples of peripheral blood. To exclude lupus and antiphospholipid syndrome as autoimmune causes of RIF, we examined lupus anticoagulant and anticardiolipin antibodies according to the protocols of a previous study [49]. We evaluated thrombophilia by testing for protein C and S deficiencies and the presence of anti-α2 glycoprotein antibodies using methods described in a previous study [50].

### Genotype analysis

Genomic DNA was extracted from whole blood using the G-DEX II Genomic DNA Extraction kit (Intron Biotechnology Inc., Seongnam, Korea). The DNA was diluted to 100 ng/μl with 1 × TE (Tris-EDTA) buffer, and then 1 μl from each sample was used to amplify polymorphisms. All PCR experiments were performed using an AccuPower HotStart PCR PreMix (Bioneer Corporation, Daejeon, Korea). Genotyping analysis was performed by polymerase chain reaction-restriction fragment-length polymorphism (PCR-RFLP) [51] analysis with previously published primers [19]. The primers and restriction enzymes are shown in Additional file 1: Table S1.

### Assessment of blood coagulation status

The platelet count (PLT), white blood cells (WBC), and hemoglobin (Hgb) levels were measured using the Sysmex XE 2100 Automated Hematology System (Sysmex Corporation, Kobe, Japan). An ACL TOP automated photo-optical coagulometer (Mitsubishi Chemical Medience, Tokyo, Japan) was used to measure the prothrombin time (PT) and the activated partial thromboplastin time (aPTT).

### Statistical analysis

Multivariate logistic regression was used to compare the differences in genotype and haplotype frequencies between the patients with RIF and the controls. Allelic frequencies were assessed for Hardy-Weinberg equilibrium (HWE) using *P* < 0.05 as the significance threshold. We used adjusted odds ratios (AORs) and 95% confidence intervals to assess the associations between different genotypes and RIF. *P* < 0.05 was considered to indicate statistically significant differences. Differences in hormone concentrations (E2, FSH, LH, PRL, and TSH) were evaluated in accordance with miRNA genotypes and alleles using independent two-sample *t* tests or one-way analysis of variance with a post hoc Scheffé test for all pairwise comparisons, as appropriate. Data are presented as the mean ± standard deviation. Statistical analyses were performed using GraphPad Prism version 4.0 (GraphPad Software, Inc., La Jolla, CA, USA) and StatsDirect version 2.4.4 (StatsDirect Ltd., Altrincham, UK).

## Supplementary information

**Additional file 1: Table S1.** Details of miRNA polymorphisms for PCR-RFLP analysis.

## Data Availability

The datasets used and/or analyzed during the current study are available from the corresponding author on reasonable request.

## Abbreviations

RIF: Recurrent implantation failure
SNP: Single nucleotide polymorphism
IVF-ET: In vitro fertilization-embryo transfer
PCR-RFLP: Polymerase chain reaction-restriction fragment-length polymorphism
OR: Odds ratio
miRNA: Micro RNA
PRL: Prolactin
TSH: Thyroid-stimulating hormone
FSH: Follicle-stimulating hormone
LH: Luteinizing hormone
E2: Estradiol
HWE: Hardy-Weinberg equilibrium
PT: Prothrombin time
aPTT: Activated partial thromboplastin time
