# Supplementary material for: Associations between microRNA (miR-25, miR-32, miR-125, and miR-222) polymorphisms and recurrent implantation failure in Korean women
Source: Hum Genomics. 2019 Dec 16;13:68. doi: 10.1186/s40246-019-0246-y (PMC6915893; doi:10.1186/s40246-019-0246-y)
Supplement: Supplementary file 1 — Additional file 1: Table S1. Details of miRNA polymorphisms for PCR-RFLP analysis. [file 40246_2019_246_MOESM1_ESM.docx]

| Additional file 1: Table S1. Details of miRNA polymorphisms for PCR-RFLP analysis | | | |
| --- | --- | --- | --- |
| Polymorphisms | rs number | Primer sequence | Restriction enzyme |
| ***miR-25*** | rs1527423 | Forward 5'- C GAG AGG CTT GGG CTA GTA A -3'  Reverse 5'- C GGA GAG GAC CAC TAT CTG C -3' | *Sac*I |
|  |  |  |  |
| ***miR-32*** | rs7041716 | Forward : 5'- G AAT CGC ATG GCA TAC ACA C -3'  Reverse 5'- A TGT TGT CAC GGC CTC AAT G -3' | *Alu*I |
|  |  |  |  |
| ***miR-125a*** | rs12976445 | Forward : 5'- C CCC TCC CGA TAT CTC TCT G -3'  Reverse 5'- C CTG GAT GTC CTC ACA GGT T -3' | *Msp*I |
|  |  |  |  |
| ***miR-222*** | rs34678647 | Forward : 5'- T TGC ACA TTT TCT TTG GAT CA -3'  Reverse 5'- T TTG TTT GCT GCT GGA TCT C -3' | *Bcl*I |
|  |  |  |  |
| PCR-RFLP, polymerase chain reaction-restriction fragment length polymorphism. | | | |
